# A 1-year longitudinal study on COVID-19 convalescents reveals persistence of anti-SARS-CoV-2 humoral and cellular immunity

**DOI:** 10.1080/22221751.2022.2049984

**Published:** 2022-03-30

**Authors:** Yang Li, Xi Wang, Xu-Rui Shen, Rong Geng, Nan Xie, Jun-Feng Han, Qing-Miao Zhang, Zheng-Li Shi, Peng Zhou

**Affiliations:** a CAS Key Laboratory of Special Pathogens, Wuhan Institute of Virology, Chinese Academy of Sciences, Wuhan, People’s Republic of China; b Anyang Municipal Center for Disease Control and Prevention, Anyang, People’s Republic of China; c University of Chinese Academy of Sciences, Beijing, People’s Republic of China

**Keywords:** SARS-CoV-2, COVID-19 convalescent, 1-year after infection, antibody durability, T cell response

## Abstract

The immune memory of over 400 million COVID-19 convalescents is not completely understood. In this integrated study, we recorded the post-acute sequelae symptoms and tested the immune memories, including circulating antibodies, memory B cell, and memory CD4 or CD8 T cell responses of a cohort of 65 COVID-19 patients over 1-year after infection. Our data show that 48% of them still have one or more sequelae symptoms and all of them maintain at least one of the immune components. The chances of having sequelae symptoms or having better immune memory are associated with peak disease severity. We did four-time points sampling per subject to precisely understand the kinetics of durability of SARS-CoV-2 circulating antibodies. We found that the RBD IgG levels likely reach a stable plateau at around 6 months, albeit it is waning at the first 6 months after infection. At 1-year after infection, more than 90% of the convalescents generated memory CD4 or CD8 T memory responses, preferably against the SARS-CoV-2 M peptide pool. The convalescents also have polyfunctional and central memory T cells that could provide rapid and efficient response to SARS-CoV-2 re-infection. Based on this information, we assessed the immune protection against the Omicron variant and concluded that convalescents should still induce effective T cell immunity against the Omicron. By studying the circulating antibodies and memory B or T cell responses to SARS-CoV-2 in an integrated manner, our study provides insight into the understanding of protective immunity against diseases caused by secondary SARS-CoV-2 infection.

## Introduction

It has been two years since the outbreak of the COVID-19 pandemic, leaving over 400 million patients as of February 2022 [1]. Following one after another waves of pandemics caused by new variants of SARS-CoV-2, particularly Delta and Omicron, whether the patients with COVID-19 recovered from the previous infection still maintain immune memory to protect themselves from severe disease caused by new variants is an important scientific question [2].

A successful COVID-19 vaccine induces good immune memory responses, including the humoral and cellular responses, which serve as protective immunity against SARS-CoV-2 infection [3]. Similarly, understanding these responses in COVID-19 convalescents is key to predict the likelihood against SARS-CoV-2 viral re-infection or secondary viral diseases [3]. In the acute phase, severe patients normally generated marked levels of SARS-CoV-2-specific antibodies and CD4^+^ or CD8^+^ T cell responses. However, compared to a strong association between the severity of disease and ineffective innate immunity, the role of adaptive responses against primary infection was still not fully understood. For example, more severe patients tend to have higher levels of neutralizing antibodies (nAbs) during primary SARS-CoV-2 infection, which contradicted to our perspective that nAbs should be protective [4]. Instead, it is well-accepted that antibody, B cell memory, and T cell memory against SARS-CoV-2 are likely important for immune protection against secondary infection [3–5]. Thus, the evaluation of these factors would sever as good indicators for the durability or efficacy of the protective immunity against diseases caused by secondary infection.

There have been pieces of evidences showing that the SARS-CoV-2 antibody levels are waning following the recovery of acute diseases [6–8]. However, an understanding of the complexities of immune memory to SARS-CoV-2 in an integrated manner is still rare, including the evaluation of antibody responses (and nAb), memory B cells, memory CD4^+^ or CD8 ^+^ T cell responses in a given population of COVID-19 convalescents. In this study, we conducted a comprehensive analysis of a group of 65 patients over a 12-month period. We recorded their peak disease symptoms (*n* = 61) and post-recovery symptoms (*n* = 50), tested the dynamic changes of antibody levels at an interval of 6-month during a year (*n* = 63), determined their SARS-CoV-2-specific memory B and T cell response at 12 months after recovery (*n* = 39), and finally predicted their protection against Omicron strains. The findings provide insight into our understanding of the protective immunity of COVID-19 convalescents against secondary infection.

## Results

### Clinical manifestation and 1-year outcome of COVID-19 patients

**Figure 1.**
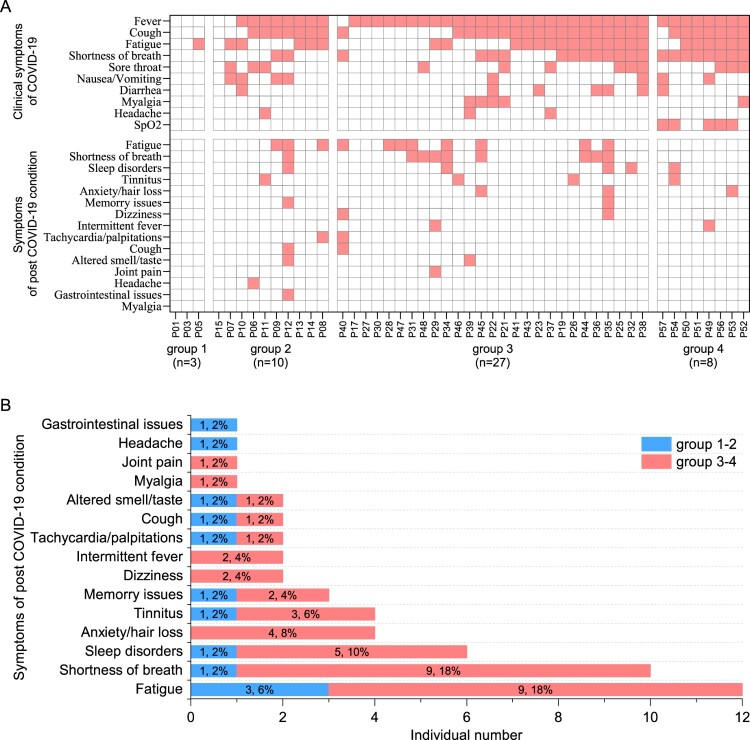
Clinical manifestation and 1-year outcome of COVID-19 patients. (A) The clinical symptoms upon admission and sequela of patients with COVID-19 at 1-year after infection (*n* = 48). (B) The sequela constitute of COVID-19 for the 13-months convalescents (*n* = 50). According to their peak disease severity, four groups were classified: asymptomatic (group 1), mild (group 2), moderate (group 3), and severe (group 4).

We recorded the peak disease symptoms and the sequelae symptoms over a year, as well as the immune memory responses to a group of 65 patients who are all from a local city Anyang in China from a single outbreak in January 2020 [9] In our cohort of COVID-19 patients, who are mostly adult above 40 years old, about 92.1% (58/63) of them experienced fever, cough, fatigue, and shortness of breath upon disease onset (Figure 1A and Supplementary Table S1). According to the severity of pneumonia, we grouped these patients into mild (group 1 and group 2) and severe (group 3 and group 4). Similar to previous reports, the disease severity is related to age [10] (Supplementary Figure 1A). Upon admission, most of the patients also experienced lymphopenia (62.3%), eosinopenia (77.0%), and increased level of erythrocyte sedimentation rate (ESR) and C-reactive protein (CRP) (more than 90%), regardless of the disease severity. We then compared the six indicators including lymphocyte count, eosinophil count, ESR, CRP, D-dimer, and procalcitonin (PCT) between the groups, and observed that all indicators are significantly worsened in the severe group (Supplementary Figure 1B-H).

We next monitored the sequelae symptoms at 1-year (13–14 months) post convalescence for 50 traceable patients. It can be observed that nearly half of the patients (24/50, 48%) still have at least one sequelae symptom (Figure 1A and Supplementary Table 2). It also appears that severe patients are more likely to have sequelae symptoms, showing a 51% (19/37) in the severe groups versus 38% (5/13) in the mild groups (Supplementary Table S2). The three major symptoms were fatigue (12/50, 24%), shortness of breath (10/50, 20%), and neurological symptoms (13/50, 26%) including sleep disorders, anxiety, tinnitus, memory issues, dizziness, and headache (Figure 1B; Supplementary Table S2). Finally, our data show that there is no association between more peak diseases and having more post convalescent symptoms (Figure 1A).

### Dynamic changes of SARS-CoV-2 circulating antibodies and the memory B cells responses at 1-year after infection

We also monitored SARS-CoV-2 specific antibody levels for the patients upon disease onset or after 3 weeks (0.75-month), 6-month and 13-month. We did consecutive sampling for 30 patients and discontinuous sampling for another 33 patients. We tested IgM, IgG, and IgA antibodies against SARS-CoV-2 receptor-binding domain (RBD), as which is the main target of nAbs. We also tested the neutralizing activity for selected serum and RBD-specific memory B cell responses at 1-year after infection.

**Figure 2.**
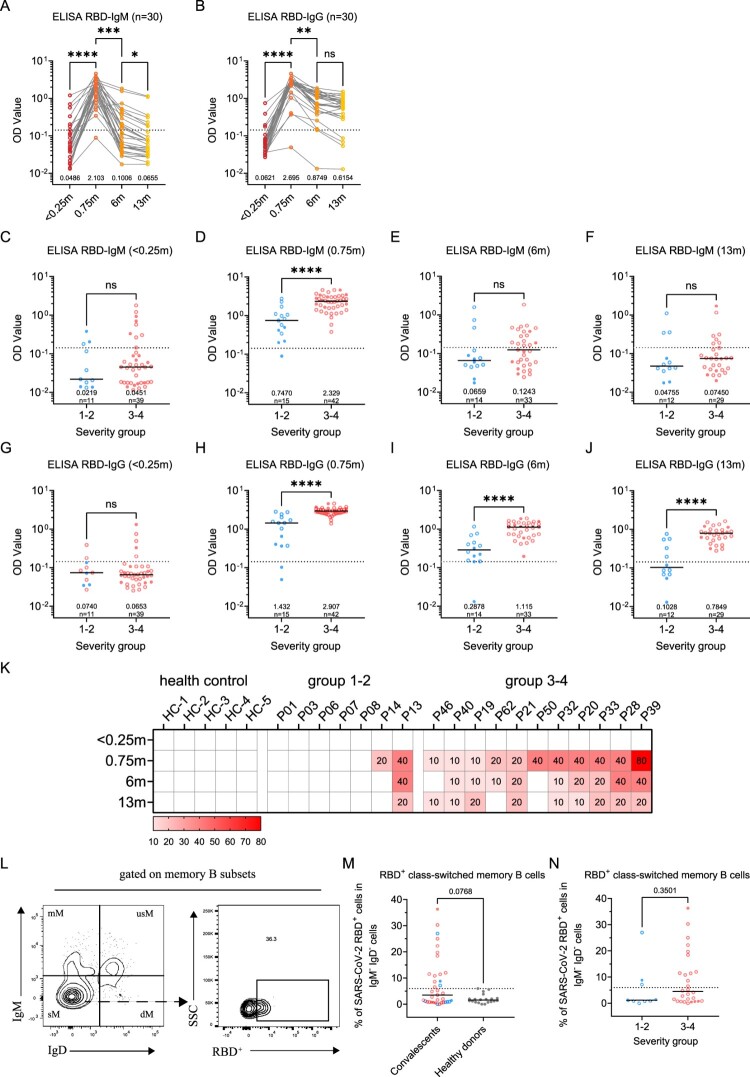
Dynamic changes of SARS-CoV-2 circulating antibodies and the memory B cells responses at 1-year after infection. Four times sampling (<0.25, 0.75, 6, and 13 m) were conducive for patients. (A-B) Continuous monitoring of the RBD-IgM and RBD-IgG levels (ELISA) in the four indicated time points based on consecutive sampling to 30 subjects. Friedman test with subsequent Dunn’s multiple comparisons was performed. The dotted line represents the cut-off value. (C-F) The RBD-IgM levels were compared between group 1–2 (milder) and group 3–4 (severer) patients, shown as data from discontinuous monitoring to all patients. Mann-Whitney test was performed. Median values and the number of subjects are shown above the X-axis. Blue-filled circles, blue-hollow circles, red-hollow circles, and red-filled circles represent the patients of groups 1, 2, 3, and 4, respectively. The dotted line represents the cut-off value. (G-J) The RBD-IgG levels were compared, similar analysis and labelling as RBD-IgM. (K) The heatmap of the neutralization antibody levels, shown as the highest dilutions that has neutralization activity. (L) The gating strategy of RBD^+^ class-switched memory B cells. (M-N) The comparisons of RBD^+^ class-switched memory B cells between convalescents and healthy donors (M), and between the mild and severe groups (N). Mann-Whitney test was performed. Grey circles represent the healthy donors; coloured circles have the same meaning as above.

Our data showed a trend of declining for all kinds of antibodies since the peak levels at 0.75-month upon disease onset (Figure 2A,B; Supplementary Figure 1M-N). In the sixth month, most patients barely have any detectable IgM or IgA antibodies, although they may still maintain a significant amount of IgG antibodies. However, the IgG antibody levels would persist from the 6-month to the 13-month, albeit they may maintain this level for a longer period. Some patients still have a significant amount of IgM responses after 6 months, possibly caused by the prolonged presence of viral antigens [11]. Thus, in contrast to our understanding of an always declining antibody response, IgG levels appear to be stable after 6 months.

Moreover, we compared the antibody dynamics between the mild and severe groups at each time point in a discontinuous analysis (Figure 2C-J and Supplementary Figure 1I-L). It can be observed that the severe group has significantly higher IgM, IgG, and IgA responses than the mild group at peak levels (0.75-month) (Figure 2D, H and Supplementary Figure 1J). The trend remains at 6-month or 13-month for IgG (Figure 2I,J) but not for IgM (Figure 2E,F). Similarly, SARS-CoV-2 nAb titres in the mild or severe groups largely reassembled the patterns of RBD-IgG ELISA, showing a decreasing trend and a higher level in severe patients (Figure 2K). A significant association between disease severity, antibody levels, and the six clinical indicators can also be determined (Supplementary Figure 1O).

The presence of memory B cell responses guaranteed quick antibody responses following re-infection. In order to understand whether there is still SARS-CoV-2 specific memory B cell response in these patients after 1-year, we detected SARS-CoV-2 RBD positive IgD^-^CD27^+^ memory B cells. As expected, unexposed healthy donors maintained an undetectable level of RBD memory B cells, whereas 15/39 (38%) of the convalescents still maintain a prominent amount of memory responses, albeit the responses didn’t show a difference between mild and severe groups (Figure 2L–N). Based on these observations, the circulating IgG humoral response to SARS-CoV-2 is likely long-lasting for most patients.

### Sustained SARS-CoV-2-specific memory CD4^+^ T cell responses in convalescents of COVID-19

SARS-CoV-2-specific memory CD4^+^ T cells were identified in 39 convalescents and 20 healthy donors, using a series of 20-mer peptides overlapping by 10 amino acids covering the S1, S2, NP, M, and E/ORFs proteins of SARS-CoV-2 (Supplementary Table S3). The viral-specific memory CD4^+^ T cells responses were measured upon quantification of IFN-γ expression after stimulation with SARS-CoV-2 peptide pools on in vitro expanded peripheral blood immune cells. IFN-γ responses of CD4^+^ T cells in convalescents and healthy donors were detected using FACS upon stimulation or not.

**Figure 3.**
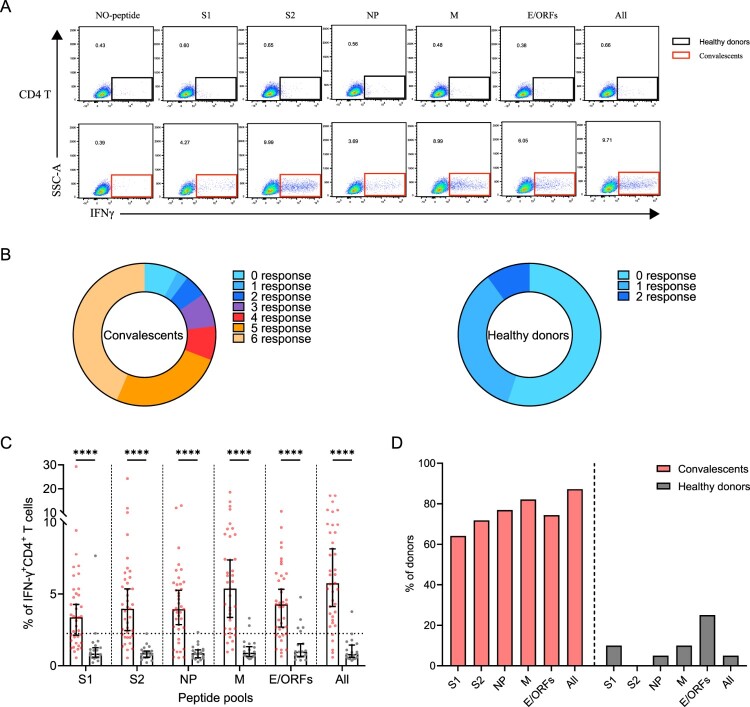
Sustained SARS-CoV-2-specific memory CD4^+^ T cell responses in convalescents of COVID-19. (A) In vitro expanded PBMCs from convalescents or healthy donors were stimulated with or without SARS-CoV-2 S1, S2, NP, M, E/ORFs peptide pools or all peptide pools for 16 h. IFN-γ producing T cells were detected by ICS assay. Flow cytometric plots representing IFN-γ-expression CD4^+^ T cells (*x*-axis) upon stimulation in the indicated convalescent or healthy donor. (B) Pie chart shows the frequency of convalescents (*n* = 39) or healthy donors (*n* = 20) who responded to 0–6 peptide pools. (C) Comparison of the relative proportion of SARS-CoV-2 peptide-pool-reactive CD4^+^ T cells between convalescents (red dots) and healthy donors (grey dots). Mann-Whitney test was performed, and bars represent median with 95% confidence interval (CI). The dotted line represents the cutoff value for absolute positive, defined as mean + 2SD of the detected frequency in no-peptide groups. (D) Percentage of convalescents or healthy donors who responded to S1, S2, NP, M, E/ORFs, or to all peptide pools.

A positive SARS-CoV-2-specific memory CD4^+^ T cells response can be determined using different kinds of peptide pools (Figure 3A). Among these individuals, 92% (36/39) of them responded to at least one peptide pool at 1-year after infection. Moreover, 77% (30/39) of convalescents also responded to more than 3 peptide pools, showing a strong CD4^+^ T memory (Figure 3B). Notably, low level (1-2 peptide pools) SARS-CoV-2-specific memory CD4^+^ T cells responses were also identified in 45% (9/20) of the unexposed healthy donors, probably caused by cross-recognition T cells specific for the common cold coronaviruses (Figure 3B) [12]. We then analyzed the viral targets and found that the all-peptides pool has the best responses (Figure 3C). Notably, the proportion of M-specific CD4^+^ T cell responses is prominently higher (32/39, 82%) than other viral targets, whereas S1 appears to be the worst target (25/39, 64%) (Figure 3C,D). These results demonstrated the general presence of long-term memory CD4^+^ T cells responses in convalescents at 1-year after infection.

### Sustained SARS-CoV-2-specific memory CD8^+^ T cell responses in convalescents of COVID-19

**Figure 4.**
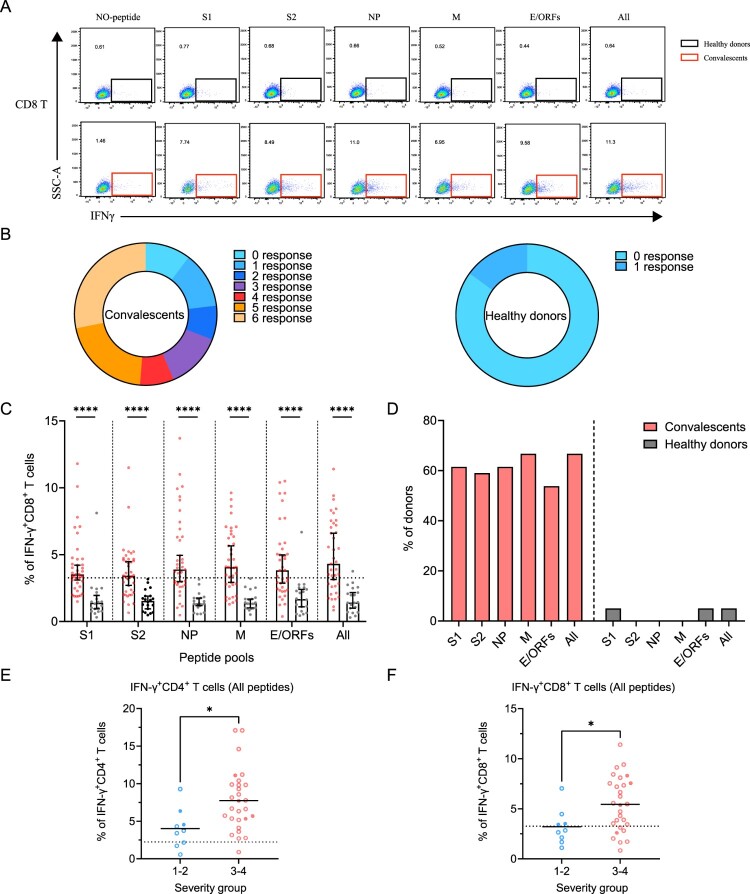
Sustained SARS-CoV-2-specific memory CD8^+^ T cell responses in convalescents of COVID-19. (A) In vitro expanded PBMCs were stimulated with or without SARS-CoV-2 S1, S2, NP, M, E/ORFs peptide pools or all peptide pools for 16 h, and the IFN-γ producing T cells were shown. (B) Pie chart shows the frequency of convalescents (*n* = 39) or healthy donors (*n* = 20) who responded to 0–6 peptide pools. (C) Comparison of the relative proportion of SARS-CoV-2 peptide-pool-reactive CD8^+^ T cells between convalescents (red dots) and healthy donors (grey dots). Statistic method and symbols are the same as in Figure 3. (D) Percentage of convalescents or healthy donors who responded to S1, S2, NP, M, E/ORFs, or all peptide pools. (E-F) The comparisons of IFN-γ^+^/CD4^+^ T cell (E) and IFN-γ^+^/CD8^+^ T cell (F) between different severity groups (n = 37, two child-convalescents were excluded). Unpaired t test was performed in these comparisons. Mean values (solid line) or cut-off (dashed line) were shown for each group. Blue-filled circles, blue-hollow circles, red-hollow circles, and red-filled circles represent the patients from groups 1, 2, 3 or 4, respectively.

Next, we determined SARS-CoV-2-specific memory CD8^+^ T cell responses using a similar strategy (Figure 4A). Similar to CD4^+^ T, 90% (35/39) of the individuals responded to at least one peptide pool. However, only 56% (22/39) of individuals responded to more than 3 peptide pools, a ratio that is lower than CD4^+^ T responses (Figure 4B). In comparison, there is only 15% (3/20) of the healthy donors responded to at least one of the peptide pools, probably due to low cross-reactivity with other common cold coronaviruses (Figure 4B). The viral targets of memory CD8^+^ T cells are also similar to CD4^+^ T cells, showing higher responses to all peptides and M-specific peptides pools compared to other pools (Figure 4C,D).

Finally, we assessed the differences in SARS-CoV-2-specific memory T cell responses between disease severity groups. As all peptide pools showed the best performance, we compared the ratio of viral-specific T cell memory between the mild and severe groups. Our data showed that either CD4^+^ or CD8^+^ memory T cells responses are significantly higher in the severe groups at 1-year after infection (Figure 4E,F).

### Polyfunctional SARS-CoV-2-specific memory CD4^+^ and CD8^+^ T cells

**Figure 5.**
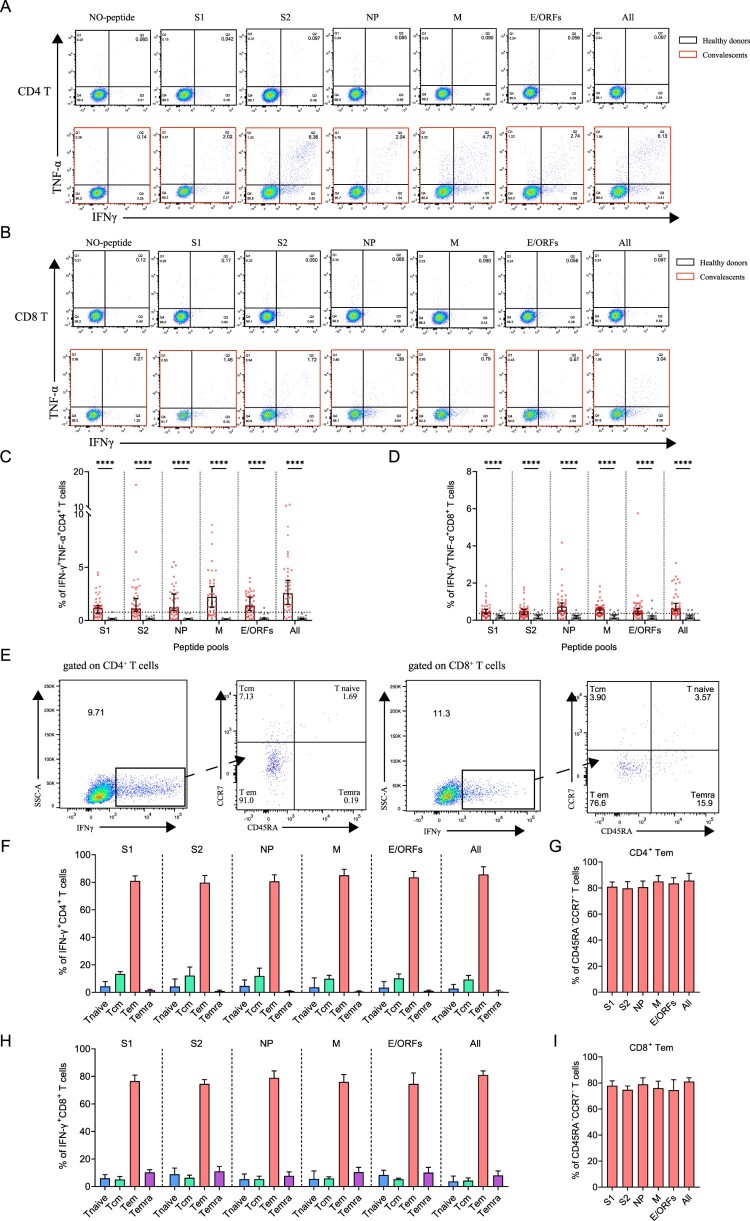
The presence of SARS-CoV-2-specific polyfunctional memory and the effector memory CD4^+^ and CD8^+^ T cells. (A-B) Gating of antigen-specific CD4^+^ and CD8^+^ T cell that are secreting both IFN-γ and TNF-α (polyfunctional T cell) after stimulation with SARS-CoV-2-specific peptide pools. (C-D) Percentage of antigen-specific polyfunctional CD4^+^ and CD8^+^ T cells in the T cell population. The statistic method and symbols are the same as in Figure 3(C) and Figure 4(C). (E) Phonotype characterization of SARS-CoV-2-specific memory CD4^+^ and CD8^+^ T cells. Cell markers used: T naïve (CD45RA^+^, CCR7^+^); central memory T cells, Tcm (CD45RA-, CCR7^+^); effector memory T cells, Tem (CD45RA^-^, CCR7^-^); and effector memory RA^+^ T cells, Temra (CD45RA^+^, CCR7^-^). (F-H) Phenotypes of antigen-specific CD4^+^ (F) and CD8^+^ T (H) cells responding to the indicated peptide pools of SARS-CoV-2 in convalescents of COVID-19. Bars are shown as median with 95%CI. (G-I) Frequency of the effector memory CD4^+^ (G) and CD8^+^ T (I) cells (CD45RA^-^, CCR7^-^) responded to SARS-CoV-2 S1, S2, NP, M, E/ORFs or all peptide pools. Bars are shown as median with 95% CI.

Polyfunctional memory T cells are expected to rapidly and efficiently respond to subsequent re-infection. To characterize if the convalescents carry multifunctional SARS-CoV-2-specific memory CD4^+^ and CD8^+^ T cells, we analyzed the cytokine-producing and phenotypic markers in T cells, including IFN-γ and TNF-α following peptide stimulation (Figure 5A,B). Our data show that the convalescents maintained both CD4^+^ and CD8^+^ polyfunctional T cells by secreting IFN-γ and TNF-α cytokines, and they had a strong CD4^+^ T response than CD8^+^ T response (Figure 5C,D). The best viral targets of polyfunctional memory CD4^+^ T cells are still the all peptides and M-specific peptide pools.

Finally, we ought to determine the phenotypes of SARS-CoV-2 specific memory T cells, which determined the types of response against re-infection. The subsets of memory T cells include naïve (CD45RA^+^CCR7^+^), central memory (CD45^-^CCR7^+^), effector memory (CD45^-^CCR7^-^) and terminally differentiated effector memory (CD45 ^+ ^CCR7^-^) cells, which can be differentiated by detecting CCR7 and CD45RA expressions on IFN-γ-producing T cells (Figure 5E) [3]. Our data show that the viral peptide pools, regardless of the types, activated the effector memory (CD45^-^CCR7^-^) CD4^+^ or CD8^+^ T cells (Figure 5F and H). This stimulation showed no significant difference between different peptide pools (Figure 5G and I). Thus, the convalescents still maintain SARS-CoV-2 specific effector memory T cells, particularly CD4^+^ T cells that could trigger T cell reactivation during a SARS-CoV-2 re-infection at 1-year after initial infection.

### Predication of the immune protection against SARS-CoV-2 Omicron variant

**Figure 6.**
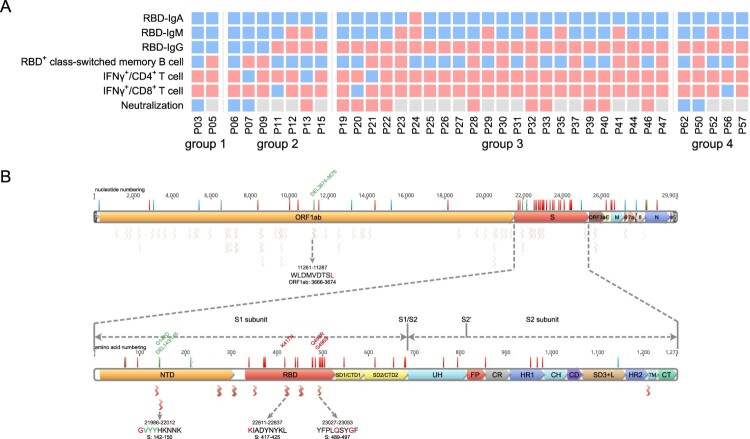
Analyzing the immune protection against Omicron variant. (A) SARS-CoV-2-specific immune memory components (*n* = 37). Pink and blue represent positive or negative responses, respectively. Grey represents no testing. (B) The conserved CD8^+^ T cell epitopes in Omicron variant. Bars with blue, red, and green on the genome represent synonymous, missense, and deletion variants respectively. Wave lines below the genome represent CD8^+^ T cell epitopes. The details of the four epitopes that contain mutations are shown.

One important issue following the quick spread of Omicron strain is whether the convalescents from the previous SARS-CoV-2 infection maintain immune protection against Omicron, which possesses the most mutations in viral genome so far [13]. We summarized various SARS-CoV-2-specific immune memory components for our cohort of convalescents and found 100% of them still maintain at least one kind of memory against SARS-CoV-2 at 1-year after infection, suggesting resistance against re-infection (Figure 6A).

We next analyzed the mutations on Omicron that may escape the humoral or T cell responses. Our data show that 15 of the key residue in the RBD region that are responsible for ACE2 neutralizing binding are mutated, suggesting a high probability of immune escaping [13]. Next, we ought to determine whether the CD8^+^ T cell response was also impaired against Omicron. In a systematic analysis of CD8^+^ T cell epitopes of SARS-CoV-2 in convalescent samples in China, 68 high-confidence conserved epitopes were identified among the Han people [14]. We found a majority of these epitopes are still conserved in Omicron, albeit the mutations in the spike region (3 epitopes) or the ORF1_ab region (1 epitope) may reduce CD8^+^ T cell responses, indicating the convalescents should still induce effective T cell immunity against the Omicron (Figure 6B).

## Discussion

Here, we recorded the post-acute sequelae syndromes and immune memory responses of a cohort of COVID-19 convalescents over 1-year after infection. Our data revealed that near half of them still have one or more sequelae symptoms, and a majority of them maintain at least one of the immune memory compartments, including antibody responses, memory B cell and memory T cell responses, which should provide protect from severe disease caused by a SARS-CoV-2 re-infection. Albeit the spike protein hyper mutations in Omicron may dampen the efficacy of neutralizing antibodies, it should have little influence on memory T cell responses.

Our data provided an integrated analysis of a given group of COVID-19 patients, from disease onset to 1-year after infection. There have been a few previous studies on the immune memory responses after SARS-CoV-2, yet most of them only provided partial information [3,4,6–8]. In an integrated analysis, we revealed that the peak disease severity is related to an elder age, a higher chance of sequelae symptoms, a higher antibody levels (including neutralizing antibodies) and a higher memory CD4 or CD8 T cell responses, thus conferring better immune protection against diseases caused by re-infection. One issue that is worrying is the persistence of sequelae symptoms over a year. However, it has been reported that the main symptoms, including dyspnoea and fatigue that are probably related to past lung injury or the use of corticosteroid therapy, are relieving in a larger cohort of COVID-19 convalescents [15]. Another issue is the waning of circulating antibody levels over time, which was thought to be associated with an increased chance of re-infection [6]. Notably, in contrast to most of the previous studies that used patient cohort rather than longitudinal data for each patient [3,6,8], our data should provide a more precise understanding of the kinetics of durability of SARS-CoV-2 circulating antibodies by doing four-time points sampling for each subject. . Thus, different to the conclusion that SARS-CoV-2 IgG responses are kept waning in previous studies, we found at least RBD IgG levels are likely to reach a stable plateau at around 6 months after infection for most of the convalescents. In contrast to short-lived declining antibodies that are produced within the 6 months after infection, the stably level of antibodies produced by long-lived memory plasma cells that have experienced affinity maturation is more effective against re-infection [3,5]. Moreover, the T cell immunity, particularly the effector memory T cells would also provide effective immune protection against re-infection.

We also realize that the protective immunity against SARS-CoV-2 is complicated, even with a thorough understanding of circulating antibodies, memory B cell, and memory T cell responses. The most straightforward test is the determination of the neutralizing antibodies, which is the only component of sterilizing immunity as revealed by serum passive transfer experiments in primates [16]. However, it can also be misleading. For example, the Omicron variant of SARS-CoV-2 possesses multiple mutations in RBD that could escape the neutralization of antibodies, raising concerns that it may cause more severe diseases [2,13]. However, the real-world clinical data suggests that Omicron is less pathogenic to humans compared to other variants, partly due to less infectivity to the lungs [17]. Yet, it is also possible that effective memory B or memory T cells induced by previous infections or by vaccination provided immune protection to Omicron patients. Moreover, the barrier immunity in the upper respiratory tract (UPT) is more important than circulating memory that is tested here against re-infection, as which is the primary target of SARS-CoV-2. Whereas circulating memory immunity may protect us from severe disease, it may not stop the re-infection if the UPT immunity (for example secreting IgA) is absent. This would be determined in future studies. Nevertheless, our data show at least one component of immune memory is still measurable in 100% of convalescents, which could provide immune protection against secondary COVID-19 diseases.

## Material and methods

### Study participants and sample collection

In the early COVID-19 epidemic of 2020, 65 patients were identified SARS-CoV-2 infection using RT-qPCR during Jan 24 to Feb 11 in Anyang city in China (ref). All of these patients were subjected to this study, which is organized by Anyang Center for Disease Control and Prevention, with written consent from all patients or their statutory guardians. From disease onset to discharge, multiple blood samples were collected, and each sample was apportioned into multiple 0.5 mL aliquots for clinical testing and −80°C storage. Moreover, follow-up studies were conducted on 47 convalescents (serum collection) at 6-month later (August 2020), or 50 convalescents (50 for sequelae investigation, 41 for blood collection) at 13-month later (March 2021). There was neither a new COVID-19 outbreak nor an initiation of a universal vaccination programme against SARS-CoV-2 in Anyang before we completed the sequelae investigation and the sample collection on March 2021. We confirmed that none of them were re-infected with SARS-CoV-2. One convalescent was excluded from this study because he received the emergency use of the COVID-19 vaccination (inactivated vaccine) between 6 and 13 months after the disease onset. The other convalescents had never been vaccinated against SARS-CoV-2 or any other pathogens during the study. Furthermore, none of the patients received vaccination in the six months prior to the infection of SARS-CoV-2. Based on the clinical characteristics of our cohort, particularly the signs of pneumonia, a severity classification including four different severity groups was determined for each patient. Patients of group 1(asymptomatic infection) had viral RNA positive, but no fever, no change of lung CT image, no obvious abnormal results in clinical blood tests. The patient of group 2 had hidden onset with mild symptoms, lung CT images showed mild and focal lesion, which could rapidly be improved in a week after admission. The patient of group 3 felt multiple discomforts, including shortness of breath, fatigue, headache, myalgia, nausea, vomiting, and sore throat between onset and admission. In addition, they also had multiple abnormal results of blood tests, and multilobar pneumonia, such as multiple patchy lesions with ground-glass opacity, which was improved after one or more than one week. Patients of group 4 presented a persistently progressed disease, such as expanding inflammation in the lungs, worsening dyspnea, and a few of them had low oxygen saturation (<93%) at rest status, and signs of type-I respiratory failure. In the following analyses, we grouped the patients into two categories, namely the milder patients (group 1-2) and the severer patients (group 3-4).

### SARS-CoV-2 specific antibody test

Antibodies binding to SARS-CoV-2 RBD were evaluated using the in-house made ELISA kits. The polystyrene 96-well plates were coated with recombinant RBD protein. 5 μL of 100-fold diluted plasma was added into wells for testing, and an HPR-conjugated mouse anti-human IgG monoclonal antibody was used to bind the antigen–antibody complex. IgM was tested using the capture-ELISA method. The polystyrene 96 well plates were coated with mouse anti-human IgMμ (second antibody). 5 μL of 100-fold diluted plasma was added into wells for testing, and an HPR-conjugated antigen was used to bind the antibody-second antibody complex. The OD value (450–630 nm) was calculated. The positive thresholds of RBD-IgM and RBD-IgG were 0.142 and 0.143, respectively. Moreover, a chemiluminescent microparticle immunoassay (CMIA) kit (Kangrun Biotech Corporation, Guangzhou, China) was used to examine RBD-IgA. The operation procedures were according to the manufacturer’s instructions. A relative luminescence value (RLV) greater than or equal to 1.0 is positive for RBD-IgA.

### Microneutralization assay with authentic SARS-CoV-2

Vero E6 cells were seeded at 1× 10^4^ cells per well into 96-well plates one day before infection. 20 μL serum was added into 180 μL DMEM of the first well, mixed and transferred 100 μL diluted plasma into the next well in which 100 μL DMEM had been added. A series of two-fold dilutions were performed from 1:10 to 1:1280. 100 μL of the virus at a working concentration (4000 TCID50/mL) was added into the diluted serum for each well, mixed, and incubated at 37°C for 1 h. An 80 μL of this virus-serum mixture was added to each Vero E6 cell well in duplication, and the wells were incubated at 37°C for 22–24 h. Cells were subsequently fixed by adding an equal volume of 70% formaldehyde to the wells, followed by permeabilization with 1% Triton X-100 for 10 min. The infection experiments were conducted in a biosafety level 3 laboratory. Immunofluorescence assay (IFA) was performed and the neutralizing titre was defined as the highest dilution of serum in which no viral positive was observed.

### SARS-CoV-2 peptide pools

The peptides were designed against SARS-CoV-2 (NC_045512) four structural (S glycoprotein, N protein, M and E proteins), and six putative accessory proteins (ORF3a, ORF6, ORF7a, ORF7b, ORF8, and ORF10) with 20-mer overlapping by 10 amino acids. All peptides were synthesized in GL Biochem Ltd. (Shanghai, China). The detailed information of the peptides was available in Supplementary Table S3.

### PBMCs isolation, in vitro culture, and T cells stimulation

The PBMCs were isolated using SepMate^TM^-50 (STEMCELL Technologies Inc., Vancouver, Canada) density gradient centrifugation according to the instructions. The blood samples were diluted with an equal volume of PBS+2%FBS and mixed gently. The density gradient medium was added to the SepMate^TM^-50 tube by carefully pipetting through the central hole of the SepMate^TM^-50. The diluted samples were centrifuged at 1200×g for 10 min at room temperature with brake. PBMCs were poured into a separate 50 mL tube and washed twice at 300×g for 10 min at room temperature with PBS+2%FBS before being cryopreserved. For in vitro culture and T cells stimulation, PBMCs were cultured for 9 days, and half of the culture medium was replaced every 3 days. The PBMCs were then stimulated at day 10 with the medium containing complete RPMI 1640 with 10%FBS, 100 U/mL penicillin, 0.1 mg/mL streptomycin (all from Gibco, USA) in the presence of 10 μM S1, S2, NP, M, E/ORFs and all peptide pools and 1 μM GolgiPlug (BD Biosciences, Franklin Lakes, New Jersey, USA) for 16 h.

### SARS-CoV-2 specific memory B cells detection

Cryopreserved PBMCs were thawed and washed twice, and 1×10^6^ PBMCs were incubated with 10 ug SARS-CoV-2 RBD S-tag protein in 100 ul PBS for 30 min at 4°C. Cells were washed twice and resuspended in FACS buffer for B cell marker staining.

### Flow cytometry

For surface staining, the following antibodies were used: FITC anti-human CD4 (RPA-T4, Biolegend (San Diego, CA, USA)), APC/Cyanine7 anti-human CD3 (UCHT1, Biolegend), Pacific Blue™ anti-human CD8a (RPA-T8, Biolegend), PE/Cyanine7 anti-human CD45RA (HI100, Biolegend), PE/Dazzle™ 594 anti-human CCR7 (G043H7, Biolegend), BB700 anti-human CD27 (M-T271, BD Biosciences), PE anti-human CD38 (HIT2, BD Biosciences), BUV737 anti-human IgD (IA6-2, BD Biosciences), APC anti-human IgM (G20-127, BD Biosciences), BV605 anti-human CD3 (SK7, BD Biosciences), BUV395 anti-human CD19 (SJ25C1, BD Biosciences). The Zombie Aqua™ Fixable Viability Kit (Biolegend) and Fixable Viability Stain 780 (BD Biosciences) were used to exclude dead cells. For intracellular cytokine staining (ICS) assay, cells were labelled for cell surface markers at 4°C for 20 min in the dark, fixed/permeabilized with Cytofix/Cytoperm Solution (BD Biosciences), and stained with intracellular antibodies, APC anti-human IFN-γ (B27, Biolegend) and PE anti-human TNF-α (Mab11, Biolegend). All samples were acquired on a BD LSR Fortessa Cytometer and data were analyzed with FlowJo version 10.8 (BD Biosciences, Franklin Lakes, New Jersey, USA).

### Statistic analysis

All statistical analyses were performed using Graphpad Prism 9 (San Diego, CA, USA) and Origin 2021 (Northampton, MA, USA). Different statistical analyses were performed according to the data type as described in figure legends. Statistical significance was set at *P *< 0.05, *P *< 0.01, *P* < 0.001, or *P* < 0.0001, as shown by asterisks.

## Supplementary Material

Supplemental Material

Supplemental Material

Supplemental Material
